# Energy Expenditure Optimization in the Echolocation of *Rhinolophus nippon*: Evidence from Heart Rate Stability

**DOI:** 10.3390/biology15120907

**Published:** 2026-06-10

**Authors:** Mingxin Zhang, Weihao Qi, Bo Han, Fujie Han, Hao Gu, Kangkang Zhang, Ying Liu

**Affiliations:** 1 Jilin Provincial Key Laboratory of Animal Resource Conservation and Utilization, Northeast Normal University, 2555 Jingyue Street, Changchun 130117, China; 2 Jilin Provincial International Cooperation Key Laboratory for Biological Control of Agricultural Pests, Northeast Normal University, Changchun 130117, China

**Keywords:** *Rhinolophus nippon*, echolocation, acoustic plasticity, heart rate, energy metabolism

## Abstract

Acoustic behavior, essential for communication and perception, is metabolically demanding. Studying the energy costs of echolocation helps us to understand animal energy allocation and provides key insights into the evolutionary constraints of acoustic signals. We examined the constant-frequency bat *Rhinolophus nippon* using a miniature electrocardiogram system and a custom servomotor that moved prey toward stationary bats. This setup allowed for synchronous recording of high-resolution electrocardiogram and echolocation calls from the search phase to the approach phase. During the search phase, bats emitted isolated echolocation pulses characterized by long pulse durations and inter-pulse intervals (IPIs), together with higher root mean square (RMS) amplitude, pulse energy, and peak amplitude. In the approach phase, call rate increased significantly (3.15-fold), and bats predominantly produced sonar sound groups. Meanwhile, pulse duration, IPIs, RMS amplitude, and pulse energy decreased to 65.23%, 25.82%, 78.50%, and 86.32% of the corresponding search-phase values, whereas peak amplitude increased to 110.99%, indicating that *R. nippon* can flexibly adjust the structure of its echolocation calls. However, despite the increased call rate (*p* < 0.05), neither heart rate nor metabolic rate differed between phases. This study provides direct physiological evidence for understanding energy expenditure in bat echolocation and offers a methodological reference for future research.

## Simple Summary

Most bats use echolocation to navigate and hunt. Research on energy consumption and regulatory strategies during vocalization helps reveal how animals manage energy to achieve an optimal balance among different behavioral investments. This balance improves animals’ survival and reproductive success. To determine how bats manage energy expenditure during intense calling, we monitored five horseshoe bats (*Rhinolophus nippon*) across 34 sessions as they detected prey using echolocation. Using miniature sensors, we simultaneously recorded the bats’ electrocardiogram signals and echolocation calls. This allowed us to measure energy expenditure during the search and approach phases. We found that as prey moved closer, though calling increased dramatically during the approach phase, heart rate and total energy expenditure remained stable compared to the search phase. This suggests that although producing calls is energetically costly, bats possess an efficient energy-saving mechanism. When call rates increase, they often use sonar sound groups (two or more pulses together) and adjust the sound features of individual pulses. These strategies keep energy expenditure stable as task demands rise. This discovery reveals a sophisticated physiological strategy shaped by evolution. It explains how bats sustain intense night-time hunting with limited energy and offers new insight into animal adaptation to environmental pressures.

## 1. Introduction

Energy serves as the currency of life, and its budget balance determines the survival and reproduction of organisms. Due to the spatial and temporal heterogeneity of resource distribution and the finite nature of individual energy acquisition, animals employ diverse behavioral and physiological strategies. These strategies reduce energy expenditure, optimize energy allocation, and improve energy utilization efficiency [1,2,3,4,5,6]. Behaviors such as locomotion for displacement, foraging to acquire essential resources, and reproduction to ensure offspring survival are all associated with substantial metabolic costs [7,8,9,10]. Moreover, the execution of these behaviors is often accompanied by vocal behavior [8,9,10,11,12]. Vocalization requires precise neuromuscular coordination and exhibits extremely low energy conversion efficiency (typically below 10%) [11], making it a highly energy-consuming behavior. This suggests that although vocalization is crucial for communication, predation, and defense, its high metabolic cost may impose significant pressure on an individual’s energy budget. Previous studies have demonstrated substantial variation in the energetic costs of vocal behaviors across different animals [12,13,14,15]. For example, the advertisement calls of anuran amphibians can reach up to 20 times the resting metabolic rate [16]. In insects, the energy expenditure of calling exceeds that of terrestrial locomotion [17]. These differences reflect adaptive divergence among animal groups in the structure of vocal organs, sound production mechanisms, and ecological demands. Therefore, investigating energy expenditure and metabolic regulation during animal vocalization through the lens of vocal behavior provides a novel perspective for a deeper understanding of the behavioral mechanisms and functional roles underlying energy metabolism in animals.

As the second most diverse order of mammals after rodents, most bats have evolved an active echolocation system to adapt to environments such as night-time and caves [18,19]. The echolocating bats emit high-frequency ultrasound waves (typically 11–212 kHz) and analyze the returning echoes to obtain information for spatial detection [19,20]. In particular, bats with constant-frequency (CF) and frequency-modulation (FM) echolocation calls (hereafter CF-FM bats) dynamically adjust their call parameters during prey capture. They shift from broad-area detection during the search phase to emitting high call rate pulses during the approach phase, enabling precise tracking of moving prey [21]. Whether this high call rate vocalization is accompanied by significant energy expenditure has long been a question in the study of echolocation behavior and physiology [22,23].

Regarding the energy expenditure of bat echolocation, existing research has focused primarily on flight conditions. Early studies by Speakman et al. (1991) on *Eptesicus fuscus* found that vocalization during flight did not incur obvious additional energy expenditure [24]. This led to the hypothesis that echolocation calls are coupled with wingbeats in the respiratory cycle, thus making them energetically free [24]. Subsequent comparative studies across bats of different body sizes have yielded similar conclusions [25]. Notably, Currie et al. (2020) reported that high-intensity echolocation imposes metabolic costs during wind tunnel flight [22]. However, flight itself entails substantial metabolic demands, which may mask the precise quantification of the independent energy expenditure of vocal behavior. Therefore, it is necessary to investigate the energy expenditure of vocalization in bats under non-flight conditions. Studies in non-flight conditions indicate that bat vocalization may exceed 1.4 times the resting metabolic rate [12], and heart rate, a proxy for energy expenditure, is closely correlated with call parameters [26,27]. Most previous studies have focused on the bats emitting frequency-modulated echolocation calls with short pulses, whereas research on bats that emit CF-FM echolocation calls with substantially longer pulses remains limited. Recent studies on the coupling between sound production and wingbeats have shown that bats can flexibly adjust the coupling relationship during flight to optimize sensory information acquisition [28]. This flexibility may also imply energetic considerations. These findings suggest that even under non-motor conditions, vocalization incurs significant physiological costs and has the potential for metabolic regulation. However, due to limitations of traditional measurement techniques, the metabolic dynamics of bat vocalization under non-flight conditions and its regulatory role remain rarely reported.

Due to the small body size of echolocating bats, previous studies often employed the doubly labeled water or open-flow respirometry systems to measure changes in energy expenditure over time [29,30]. In recent years, heart rate has been widely used as an effective proxy for energy metabolism in vertebrates, including bats [3]. Heart rate monitoring techniques have gradually become miniaturized and multifunctional [31], enabling sensor-based measurements of locomotor energy expenditure and providing a convenient approach to assess echolocation energy costs over specific time periods, with continuous heart rate data [32]. *Rhinolophus nippon* (hereafter *R. nippon*) is a typical CF-FM bat; its relatively large body size satisfies the requirements for attaching metabolic monitoring devices, and it has a well-established ecological and behavioral research background [33,34,35], making it an ideal model for studying the plasticity of echolocation vocalization and energy metabolism.

In this study, *R. nippon* were presented with prey approaching at a constant speed by using a servo-driven system. Echolocation calls and high-temporal-resolution electrocardiogram signals were recorded synchronously from the search to approach phases. By comparing echolocation acoustic parameters across different vocal phases and analyzing their relationship with real-time metabolic rate derived from heart rate, we investigated the strategies by which *R. nippon* regulates vocalization and energy expenditure in a non-flight state. We hypothesize that *R. nippon* can flexibly adjust the spectrum-temporal parameters of its echolocation calls during prey approach, with concomitant regulation of energy expenditure. This study provides direct physiological evidence for understanding the energy expenditure of echolocation in bats and offers a new perspective for the broader field of energy ecology of animal acoustic behavior.

## 2. Materials and Methods

### 2.1. Study Animals and Domestication

Five adult individuals (two males and three females) of the greater horseshoe bat, *R. nippon*, were used in this study. Adulthood was determined based on forearm length, fur color, epiphyseal cartilage closure status, and body weight. Each bat was assigned an identification number from Rf 1 to Rf 5. They were collected in 2024 from Ji’an City, Jilin Province, China. All experimental individuals were in good health and non-pregnant. The body mass of the bats was maintained at a consistent level throughout the experiment.

Bats were housed in a laboratory room maintained at 23–26 °C with relative humidity controlled between 50% and 65%. The room was equipped with a ventilation system to ensure hygienic conditions. The light cycle was set to 12 h light/12 h dark, and bats were fed fresh mealworms daily. All bats underwent at least 7 days of acclimation, and the formal experiment commenced only after they demonstrated voluntary feeding and drinking, regular circadian activity, and no significant body mass loss. No bats died during the experimental period. After the experiments, all bats were released back to the collection cave in good health.

### 2.2. Echolocation Calls and Electrocardiogram Recording

During the experiments, electrocardiogram signals and echolocation pulses emitted by the bats were recorded using a physiological and motion sensor (Vesper Pipistrell, VT04-PP, A.S.D., Haifa, Israel). This sensor consists of a mainboard chip, an inertial measurement unit (IMU) sensor board, and an electrocardiograph (ECG) sensor board, with a total weight of 3.3 g. The microphone module on the mainboard works in coordination with the other modules to record real-time, synchronized ECG signals and echolocation pulses. This setup enables high-precision alignment between the ECG signals and echolocation pulses. After the sensor was connected to a computer for time configuration, all three modules began working simultaneously, ensuring precise temporal correspondence between acoustic and ECG signals. Echolocation signals were sampled at 250 kHz, and ECG signals were sampled at 500 Hz. The setting of the sensor is achieved by modifying the parameters of the configurable size in the program config file of the sensor’s app.

Prior to the experiments, the dorsal fur of each bat was shaved. Pure silver sensor electrodes (1 mm in diameter) were implanted and fixed using 3M veterinary tissue adhesive, without causing bleeding. The microphone module of the sensor was positioned facing the bat’s head at a distance of 1 cm. The sensor was activated by gently swiping a magnet over its induction area, allowing it to start without physical contact.

### 2.3. Experimental Setup and Prey Approach Simulation

To investigate the energetic costs and acoustic changes of bat echolocation from the search to approach phases, while eliminating the influence of high-intensity locomotion (i.e., flight) on energy metabolism, we designed a servo-driven experimental apparatus to move prey toward stationary bats. This setup elicited echolocation calls, simulating the natural process of prey approach. Experiments were conducted in a professional acoustic laboratory. All four walls were covered with thick sound-absorbing foam to minimize echoes and noise interference. To minimize disturbance to the bats’ natural behavioral rhythms, all acoustic recordings and ECG measurements were performed during their active period, from 15:00 to 22:30.

**Figure 1:**
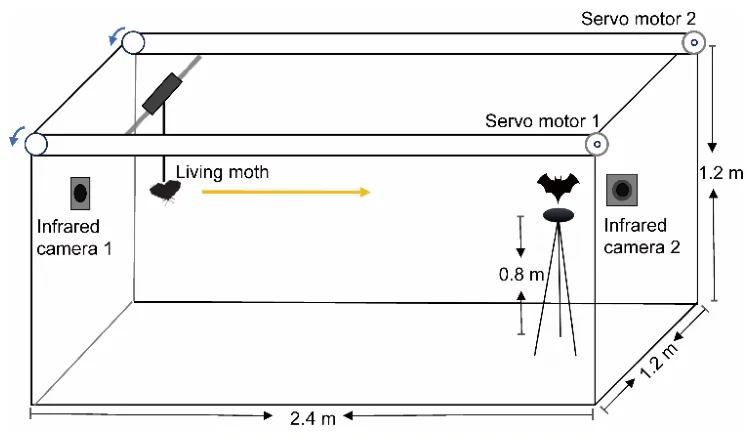
Schematic diagram of the experimental setup. The blue curved arrow indicates the direction of the conveyor belt driven by the motor, and the yellow arrow shows the direction of prey movement. During the experiment, the prey moved in a straight line at constant speed toward the bat, which remained in a fixed position.

Prey moving toward stationary bats was facilitated by a custom-built motor system comprising two servo motors. These motors rotated to drive a conveyor rod carrying flapping moths (*Spodoptera litura*) gradually and at a constant speed (Figure 1). The prey speed and trajectory were controlled by the host computer, which set the target position and velocity profile via the controller interface. Each individual was exposed to the same experimental procedure, and repeated measurements were accounted for statistically by including Bat ID as a random effect in the mixed-effects models.

Bats were trained to stay on a stable platform, and all experiments were conducted in complete darkness to eliminate visual cues. During the experiment, bats were gently wrapped in towels and secured on a foam platform at the opposite end of the servo-driven system, with their sensors fully exposed. The resting platform was positioned 0.8 m above the ground. Sensors worn by the bats simultaneously recorded ECG signals and echolocation calls throughout the experiments. Two infrared cameras (Sony HDR PJ760E, Sony, Tokyo, Japan) were used to record the bats’ echolocation behavior during the experiments. In addition, an ultrasonic detector (UltraSoundGate 116, Avisoft Bioacoustics, Berlin, Germany) was placed 0.8 m from the bat on the ground, with the microphone facing the bat’s head to record high-quality echolocation calls. These recordings complemented those collected by the physiological and motion sensors for subsequent detailed acoustic analyses. The device has a frequency response range of 20 Hz to 460 kHz, covering all harmonic components of the bats’ calls. The input sensitivity is adjustable (−43.2 dBV to −3.2 dBV), ensuring that weak echoes can be recorded clearly at a distance of 1 m while preventing signal overload from strong calls. Recordings were sampled at 375 kHz with 16-bit resolution, in accordance with the Nyquist sampling criterion.

Each experimental trial lasted 30 s to provide sufficient detection time for the bats. The motor drove the prey at a constant speed of 0.1 m/s toward the stationary bat for 2.0 m, and then remained in front of the bat for 10 s. To prevent habituation, each bat was tested every 30 min. Experiments were conducted during the bats’ active period, with laboratory lights turned off to maintain complete darkness. Temperature and humidity were recorded during the experiments using a thermo-hygrometer. A total of 34 experimental trials capturing natural variation in echolocation calls were obtained.

Prior to the vocalization experiments, the motor was operated to drive the conveyor belt without a moth attached. No obvious responses or echolocation calls from the bats were observed during motor operation, indicating that the motor apparatus did not elicit sustained vocalization from the bats.

### 2.4. Echolocation Pulses Analysis

#### 2.4.1. Division of the Echolocation Phase

Bat echolocation calls were analyzed using the acoustic analysis software Avisoft SASLab Pro version 5.2 (Avisoft Bioacoustics, Berlin, Germany), focusing on the second harmonic with the highest energy. Acoustic parameters were extracted using spectrograms, Hamming windows, oscillograms, and energy spectrum diagrams. Fast Fourier Transform (FFT) was applied with a window length of 512 points. Acoustic parameters were calculated on a per-pulse basis.

**Figure 2:**
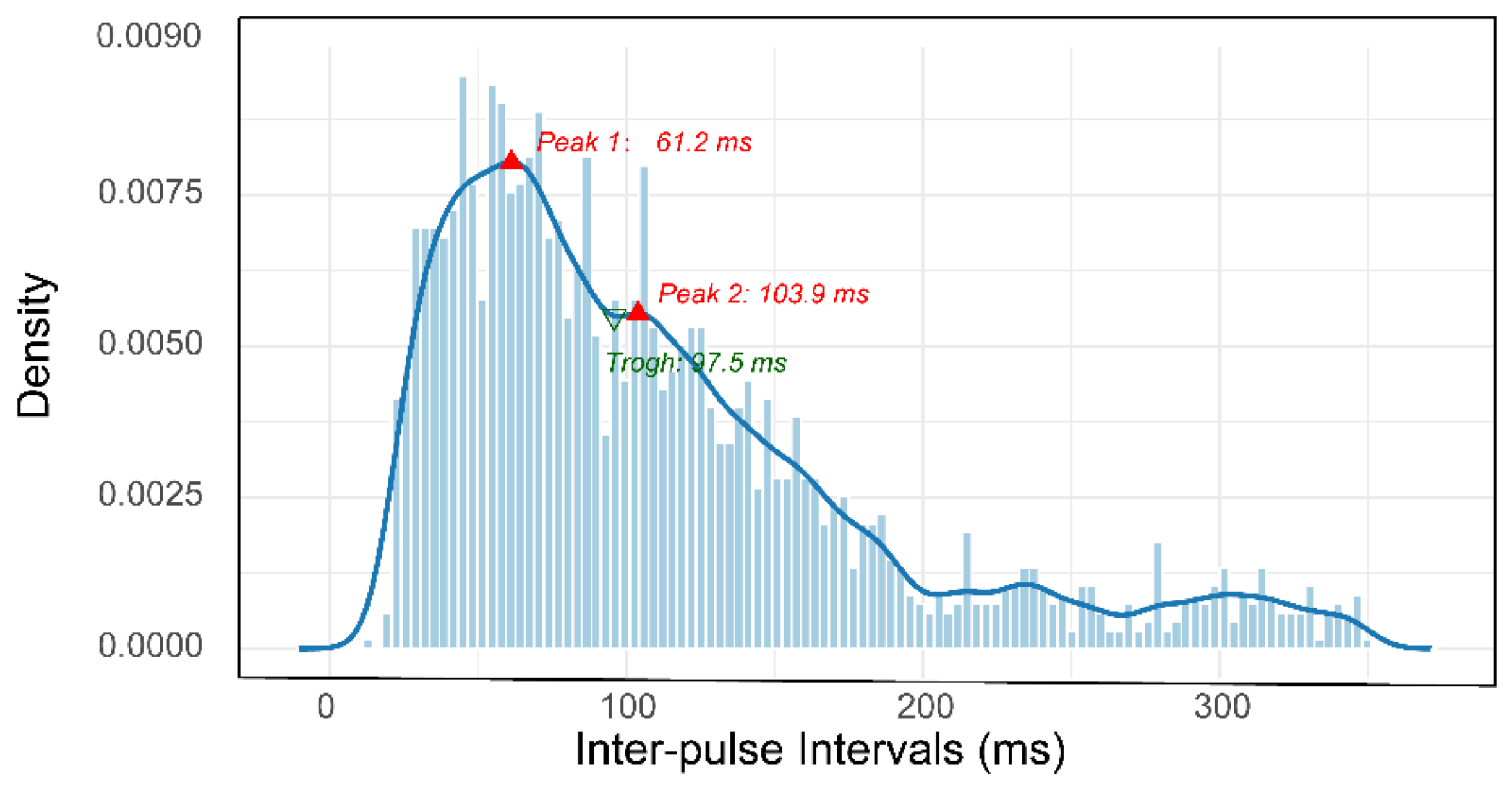
Density distribution of inter-pulse intervals in echolocation pulses of *R. nippon*. Red solid triangles represent the two peaks of the IPIs (inter-pulse intervals) bimodal distribution, while the green hollow triangle represents the trough of the pulse interval time bimodal graph.

In Python, a density distribution plot was generated based on all 3669 inter-pulse intervals. The valley of 97.5 ms was used as a threshold (Figure 2) to classify the bats’ echolocation sequences into search and approach phases.

#### 2.4.2. Analysis of Echolocation Pulse Spectral Parameters

High-quality echolocation calls were selected for analysis of acoustic spectral parameters. The parameters of interest included pulse duration, inter-pulse interval (IPI), root mean square amplitude (RMS amplitude), energy, mean peak amplitude (peak amplitude), and call rate. RMS amplitude represents the average amplitude of the call pulses, whereas energy represents the total acoustic energy within a selected time window. Without calibration, measurements of energy, RMS amplitude, and spectral peak are expressed as relative values on a logarithmic scale, using an arbitrary reference unit (digital full scale = 1). The resulting values are negative and reflect relative signal intensity. These values are commonly used for internal comparisons within the same recording system and under the same parameter settings. Because the energy values were small, they were log-transformed (base 10) for analysis. Under uncalibrated conditions, energy is treated as a dimensionless quantity. Call rate was calculated separately for the search phase and the approach phase, as the ratio of the total number of pulses to the total vocalization duration within each phase.

For echolocation spectral analysis, three high-quality echolocation calls were randomly selected from three trials for each individual, covering both the search and approach phases. In total, 456 pulses from the search phase and 1517 pulses from the approach phase were analyzed. Pulses in the approach phase mainly occurred in SSGs, including 346 doublets, 147 triplets, and 33 multiplets.

### 2.5. ECG-Based Estimation of Heart Rate, Metabolic Rate, and Oxygen Consumption

#### 2.5.1. Electrocardiogram Signal Processing and Heart Rate Calculation

The cardiac electrical signals collected during the experiments were stored as time-series files on a computer. The processing of the original electrocardiogram signal is accomplished by using median filtering in Python to remove spike noise and by using high-pass filtering to eliminate baseline drift. And within a 2 s sliding window, the find peaks algorithm is used to detect by adopting an adaptive threshold (with a maximum value of 25% of the window) and a minimum peak distance constraint. Finally, the overlapping window results are merged and duplicates are removed to obtain the R-wave peak sequence. The electrocardiogram (ECG) signals were processed according to the following steps:

(1) Signal Preprocessing

Baseline correction was first applied to the raw ECG signals using a baseline fitting method. This removed low-frequency noise caused by respiration or slow sensor drift. Subsequently, digital filtering was performed sequentially: a high-pass filter (cutoff frequency 90 Hz) was used to remove high-frequency electromyographic noise, followed by a band-stop filter to eliminate power line interference. All preprocessing steps were implemented in Python v3.13.1.

(2) R-wave Detection

The preprocessed signals were analyzed using algorithms in the HeartPy toolbox in Python to automatically identify and mark R-wave peaks. A file containing the timestamps of each detected R-wave was automatically saved. This file was then imported into the physiological signal analysis software Physiozoo (version 1.4.4) for further processing. The software first automatically removed anomalous R-wave markers resulting from false positives or missed detections based on preset thresholds (RR max = 0.24, RR min = 0.05). The average inter-beat interval was then calculated from valid, consecutive inter-beat intervals.

(3) Heart Rate Calculation

Heart rate was calculated using the following formula:

(1) $$HR\;(beats/min)=\frac{60,000}{{NN}_{mean}}$$

where *HR* is the mean heart rate (bpm), and *NN*_mean_ is the mean inter-beat interval (ms). The factor 60,000 converts milliseconds to minutes.

Heart rate calculated from the above formula was used to compare the search and approach phases. Instantaneous heart rate data for time-related analyses were obtained from Physiozoo. Measurements were recorded at approximately 0.1 s intervals and smoothed using a five-point sliding window.

#### 2.5.2. Calibration of Metabolic Rate and Oxygen Consumption

The real-time oxygen consumption rate of bats was derived from heart rate using Formula (1) and the heart rate–oxygen consumption conversion equation [36]. The oxygen consumption rate was calculated using the following formula:

(2) $$\dot{V}o_{2}=0.000476{\times M}_{b}^{1.196\pm 0.015}\times f_{h}^{2.090\pm 0.03}$$

where $\dot{V}o_{2}$ is the oxygen consumption rate (mL·min^−1^), *M*_b_ is the bat’s body mass (kg), and *f*_h_ is the heart rate (bpm). The Delta error propagation model was used to incorporate the uncertainty of the exponent into the final standard deviation calculation [37].

Oxygen consumption rate $\dot{V}o_{2}$ was converted into energy expenditure (kJ) using the oxycalorific equivalent (kJ·L^−1^), according to the following equation from Lighton [18,38]:

(3) $$MR=\dot{V}o_{2}\times [16+5.164\times \left(RER\right)],\mathrm{RER}=\;\frac{{\dot{V}co}_{2}}{{\dot{V}o}_{2}}$$

*MR* represents the metabolic rate, and the expression in square brackets calculates the oxycalorific equivalent. The oxycalorific equivalent represents the amount of energy produced per liter of oxygen consumed. *RER* (Respiratory Exchange Ratio) is the ratio of respiratory exchange, and $\dot{V}o_{2}$ represents the rate of carbon dioxide production. The constant 16 represents metabolic substrates from fat and protein oxidation, and 5.164 is a correction coefficient related to carbohydrate metabolism. Based on Speakman’s study in insectivorous bats [12], *RER* was set to 0.77. Accordingly, the oxycalorific equivalent used in this study was 19.98 kJ·L^−1^.

### 2.6. Statistic Analysis

A linear mixed-effects model (LMM) was used to test for differences in heart rate between the search and approach phases during echolocation vocalization. LMMs were fitted using the lmer() function in the lme4 package (version 2.0.1). Statistical significance of fixed effects was assessed using the lmerTest package (version 3.2.1), with denominator degrees of freedom and corresponding *p*-values estimated using the Satterthwaite approximation. The model included phase as a fixed effect, heart rate as the response variable, and Bat ID as a random intercept to account for repeated measurements from the same individual. Separate LMM analyses were also conducted to examine whether acoustic parameters of echolocation calls differed between phases. Response variables included pulse duration, inter-pulse intervals, RMS amplitude, energy, and peak amplitude. Phase was included as a fixed effect, and Bat ID was included as a random intercept.

For analyses not conducted using LMMs, data were first evaluated for normality and homogeneity of variance. Data meeting the assumptions of normality and homogeneity of variance were analyzed using one-way analysis of variance (ANOVA), followed by Scheffé post hoc tests. Data that violated these assumptions were analyzed using the Kruskal–Wallis H test, followed by pairwise Mann–Whitney U tests with Bonferroni correction.

Data preprocessing, signal extraction, and visualization were conducted in Python 3.13.1. All statistical analyses were performed in R 4.4.0.

## 3. Results

### 3.1. Characteristics and Adjustment Strategies of Bat Echolocation Calls

**Figure 3:**
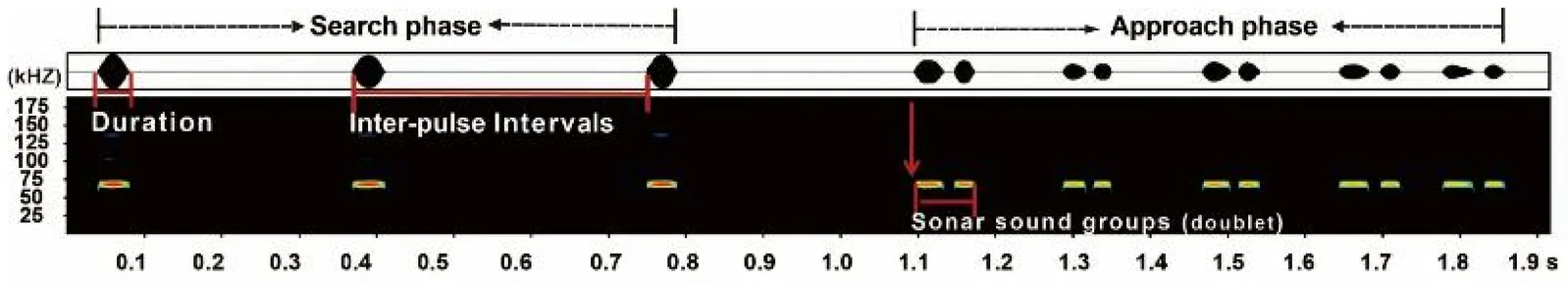
Time-domain waveform diagrams (**top**) and spectrograms (**bottom**) of echolocation calls emitted by *R. nippon* during the search and approach phases.

Echolocation call data was obtained from a total of five adult individuals (Rf 1 to Rf 5) across 34 vocalization trials, with a total recording duration of 1054 s. Echolocation calls were classified into the search phase and approach phase based on the bimodal distribution of inter-pulse intervals. During the first 0–13 s of each trial, most vocalizations occurred in the search phase, characterized by isolated pulses with relatively long pulse durations and inter-pulse intervals (Figure 3). Bats continuously adjusted head orientation, as well as the shape and orientation of their auricles, to optimize echo reception. Pulse duration and inter-pulse intervals were relatively long, averaging 38.22 ± 7.92 ms and 229.69 ± 142.67 ms, respectively. The mean RMS amplitude was −18.68 ± 12.57 dBFS, the mean log-transformed energy was −3.29 ± 1.29, and the mean peak amplitude was −25.38 ± 14.00 dBFS. During 13–30 s of the trials, *R. nippon* transitioned to the approach phase, during which bats predominantly emitted SSGs with both short pulse durations and short inter-pulse intervals. Compared with the search phase, pulse duration and inter-pulse intervals were significantly shortened (Figure 3). In the approach phase, pulse duration averaged 24.93 ± 7.06 ms, inter-pulse intervals averaged 59.29 ± 36.46 ms, RMS amplitude averaged 22.70 ± 10.67 dBFS, log-transformed energy averaged −3.74 ± 1.18, and peak amplitude averaged −22.59 ± 9.95 dBFS.

**Figure 4:**
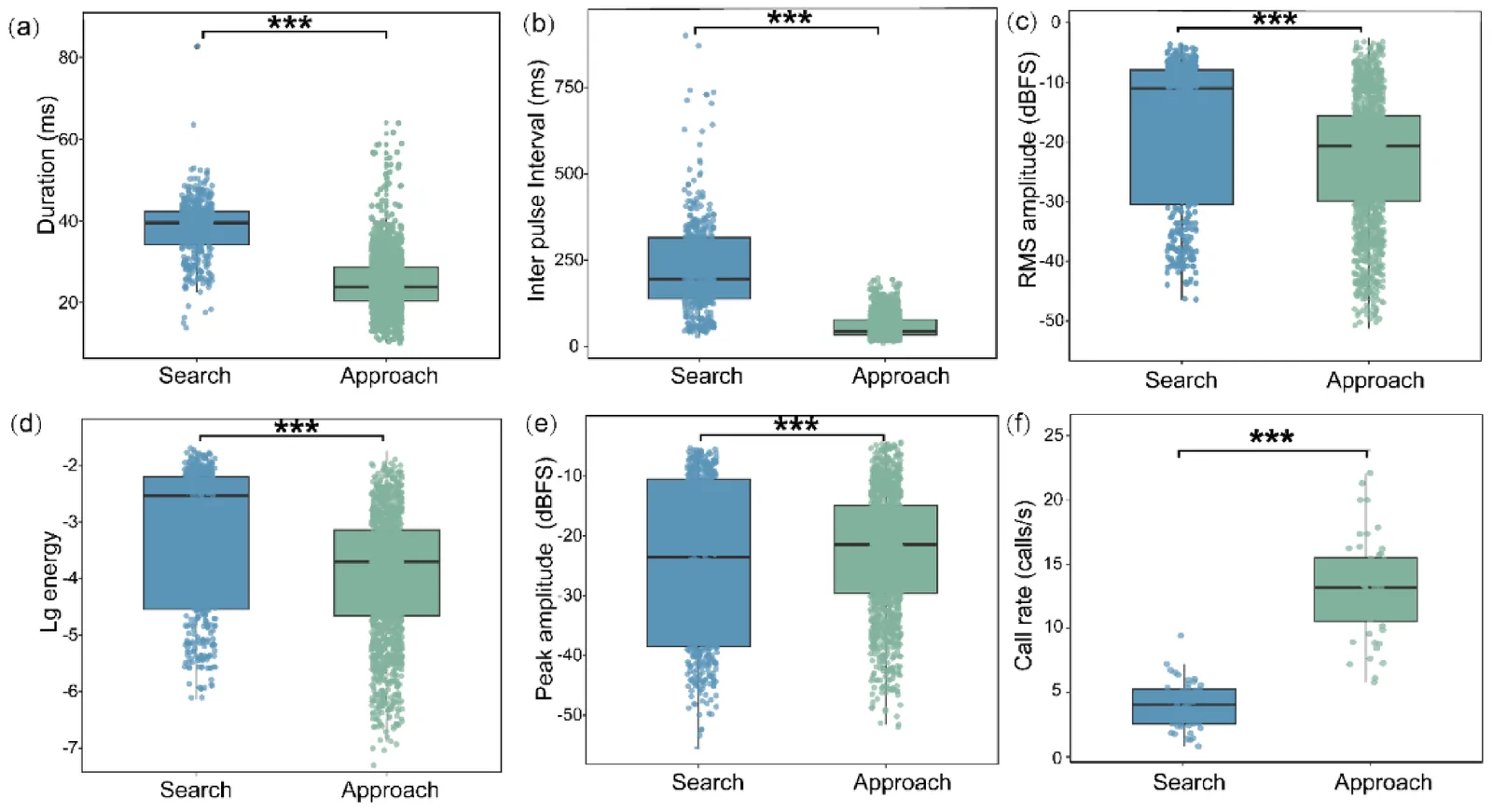
Comparison of acoustic spectral parameters during echolocation search and approach phases of *R. nippon*. (**a**) Duration of echolocation pulses; (**b**) Inter-pulse intervals (IPIs) between echolocation pulses; (**c**) Root mean square (RMS) amplitude of echolocation pulses; (**d**) Log-transformed values of pulse energy; (**e**) Peak amplitude of echolocation pulses; (**f**) Call rate. Search represents the search phase, and approach represents the approach phase. The asterisk indicates statistical significance (*** *p* < 0.001).

As the distance between the bat and the prey decreased, the call rate of *R. nippon* during the approach phase increased significantly (*p* < 0.05; Figure 4f; Table A1). The average call rate was 4.08 ± 0.27 calls/s during the search phase and 12.87 ± 0.58 calls/s during the approach phase. The call rate during the approach phase was approximately 3.15 times that of the search phase, representing an average increase of 9.01 pulses per second.

After controlling for individual differences using linear mixed models, all spectral parameters differed highly significantly between the search and the approach phase (*p* < 0.001; Figure 4), although one individual (Rf 2) only exhibited a significant difference (*p* = 0.029; Table A1). Specifically, from the search phase to the approach phase, pulse duration and IPIs decreased significantly, and the relative intensities of both the RMS amplitude and the energy after logarithmic conversion became more negative, representing a reduction in absolute relative intensity (Table A2 and Table A3). During this process, pulse duration decreased by an average of 13.29 ms, IPIs decreased by an average of 170.40 ms, RMS amplitude decreased by an average of 4.02 dBFS, log-transformed energy decreased by an average of 0.45, and peak amplitude increased by an average of 2.79 dBFS, while call rate increased significantly. The corresponding spectral parameters changed to 65.23%, 25.82%, 78.50%, 86.32%, 110.99%, and 315.44% of their values in the search phase, respectively.

**Figure 5:**
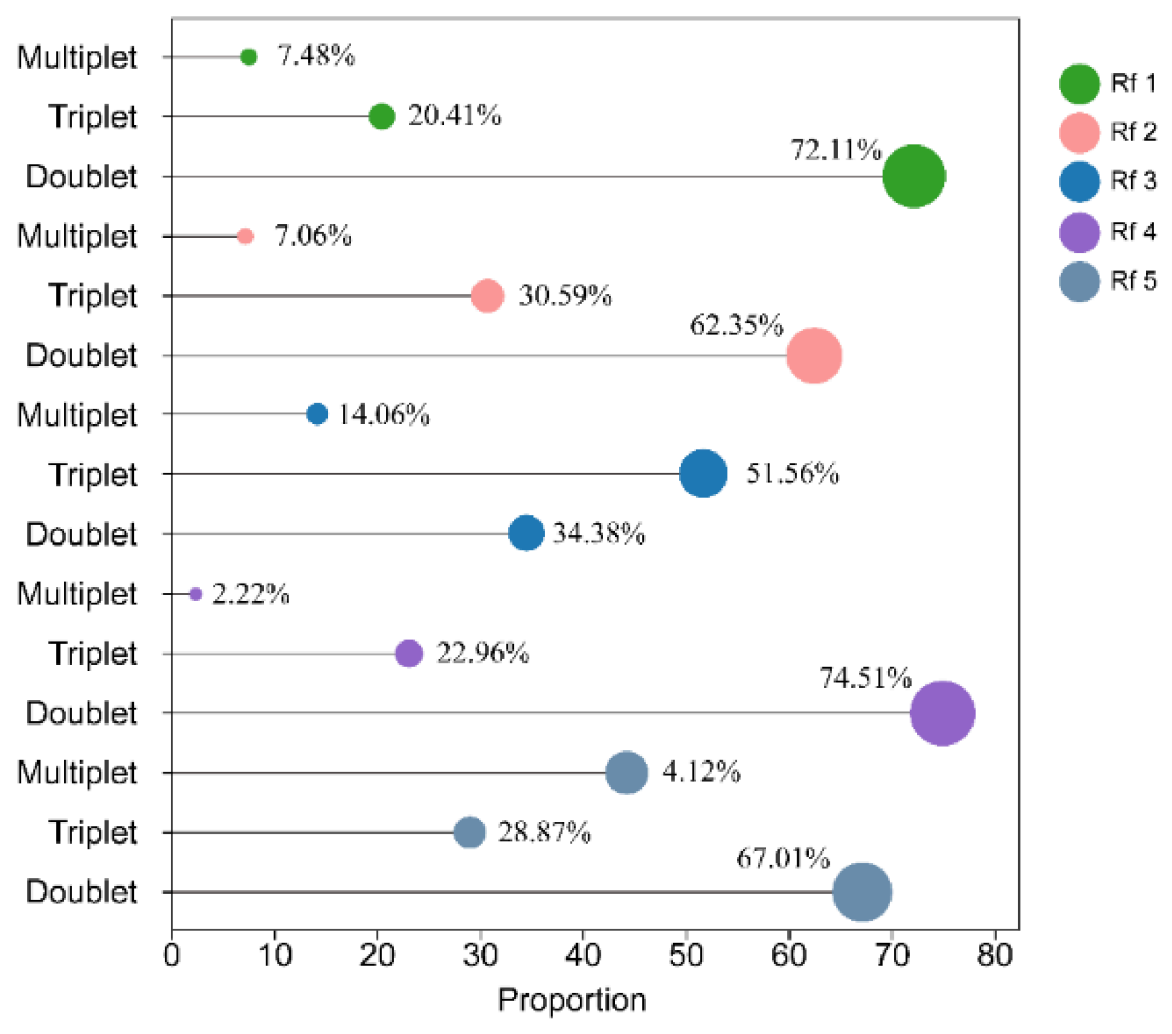
Composition of sonar sound groups (SSGs) during the approach phase of *R. nippon*.

**Figure 6:**
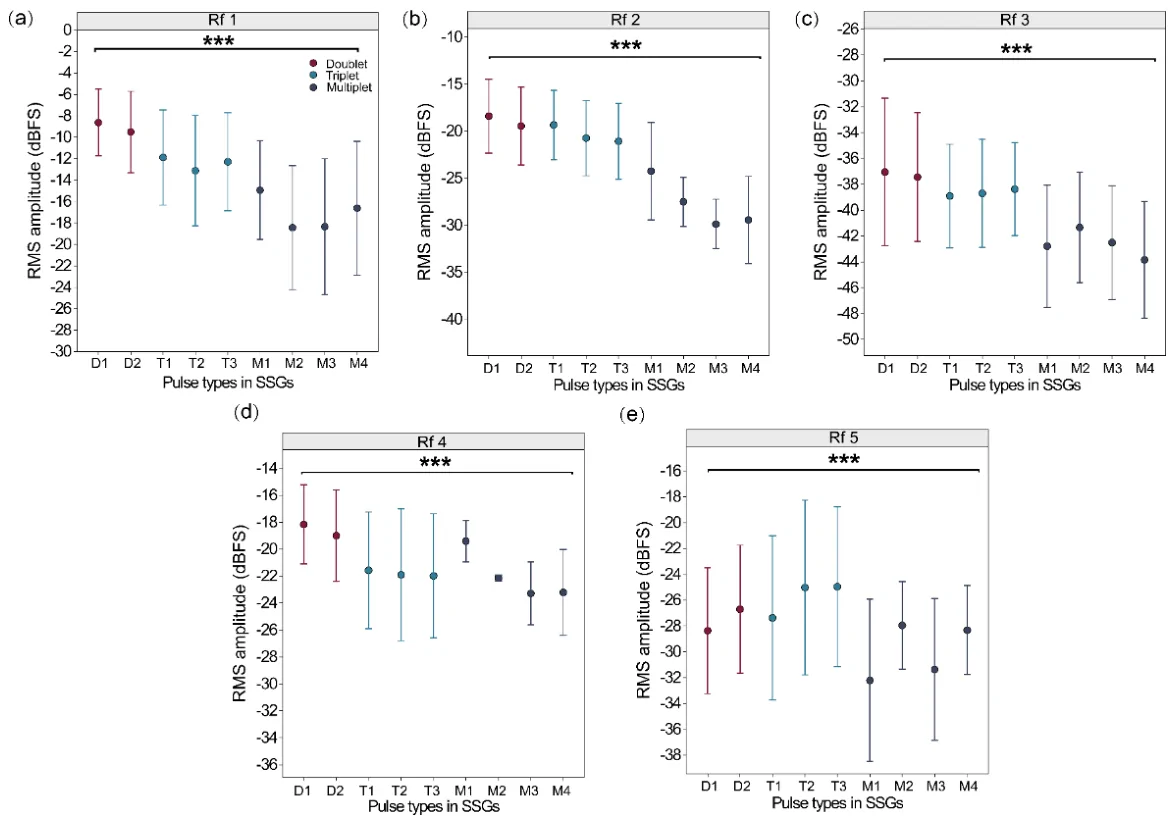
Comparison of RMS amplitudes within echolocation pulses of *R. nippon*. Panels (**a**–**e**) correspond to Rf 1–5. Asterisks indicate statistical significance (*** *p* < 0.001).

**Figure 7:**
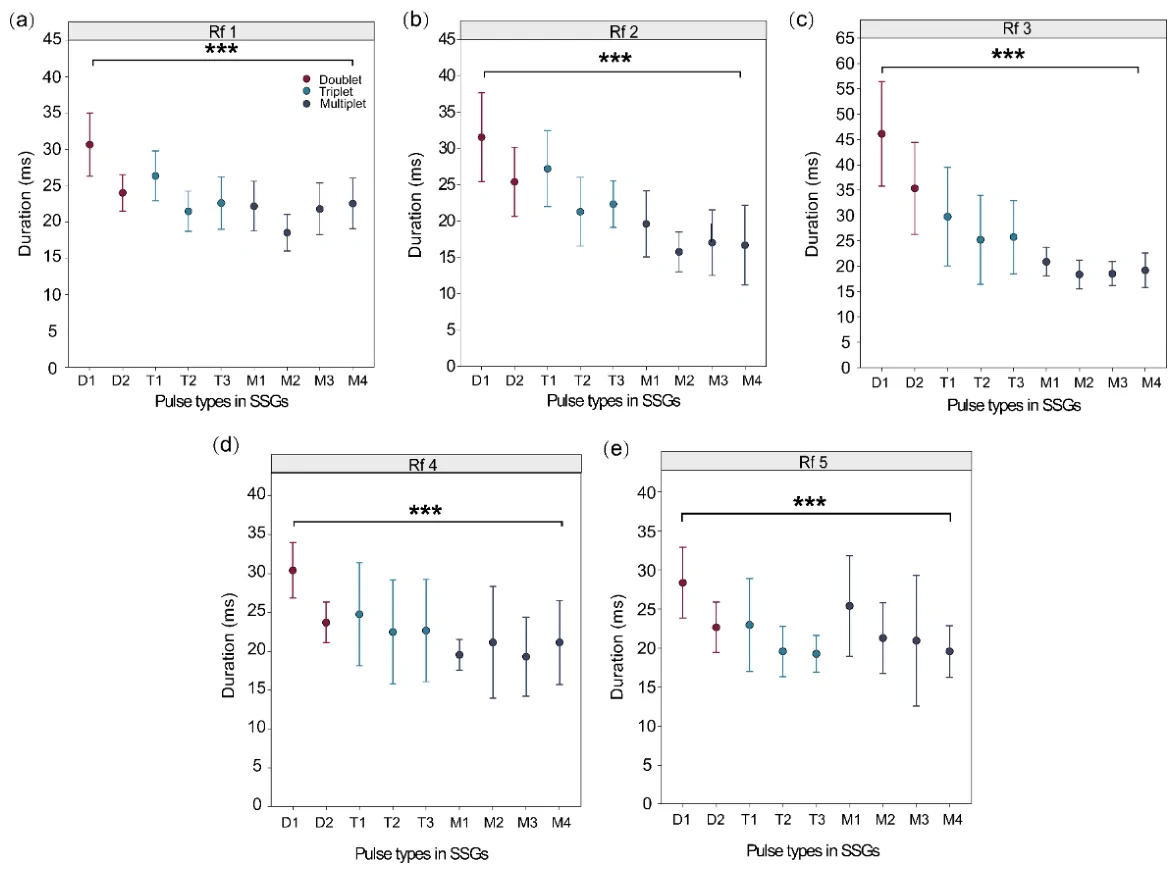
Comparison of pulse duration within the echolocation vocalizations of *R. nippon*. Panels (**a**–**e**) correspond to individuals Rf 1–5. Asterisks indicate statistical significance (*** *p* < 0.001).

Unlike the single pulses emitted during the search phase, *R. nippon* predominantly produced echolocation calls in the approach phase as SSGs composed of multiple short pulses (Figure 3). SSGs were categorized based on the number of pulses they contained: doublet, triplet, and multiplet. Since SSGs with more than four pulses were rare (less than 5% of recordings), the analysis of approach-phase SSGs focused primarily on doublet, triplet, and multiplet SSGs containing up to four pulses. Results showed that approach-phase echolocation SSGs of *R. nippon* were predominantly composed of doublet and triplet SSGs (Figure 5, Table A4). To investigate the modulation of echolocation pulses, pulse parameters were compared within SSGs for each bat. The results indicated significant differences in pulse duration and RMS amplitude among pulses within different SSGs. Specifically, pulses within doublets exhibited significantly higher RMS amplitude and longer duration than those in triplet and multiplet groups (*p* < 0.001; Figure 6 and Figure 7; Table A4), suggesting that bats can flexibly and precisely adjust pulse parameters during vocalization.

### 3.2. Heart Activity and Energy Metabolism During Echolocation Vocalization in R. nippon

In this study, high temporal resolution electrocardiogram (ECG) signals from five individuals across 34 trials were used for heart rate and metabolic rate analysis. During echolocation vocalization, all five experimental bats exhibited high heart rates.

**Figure 8:**
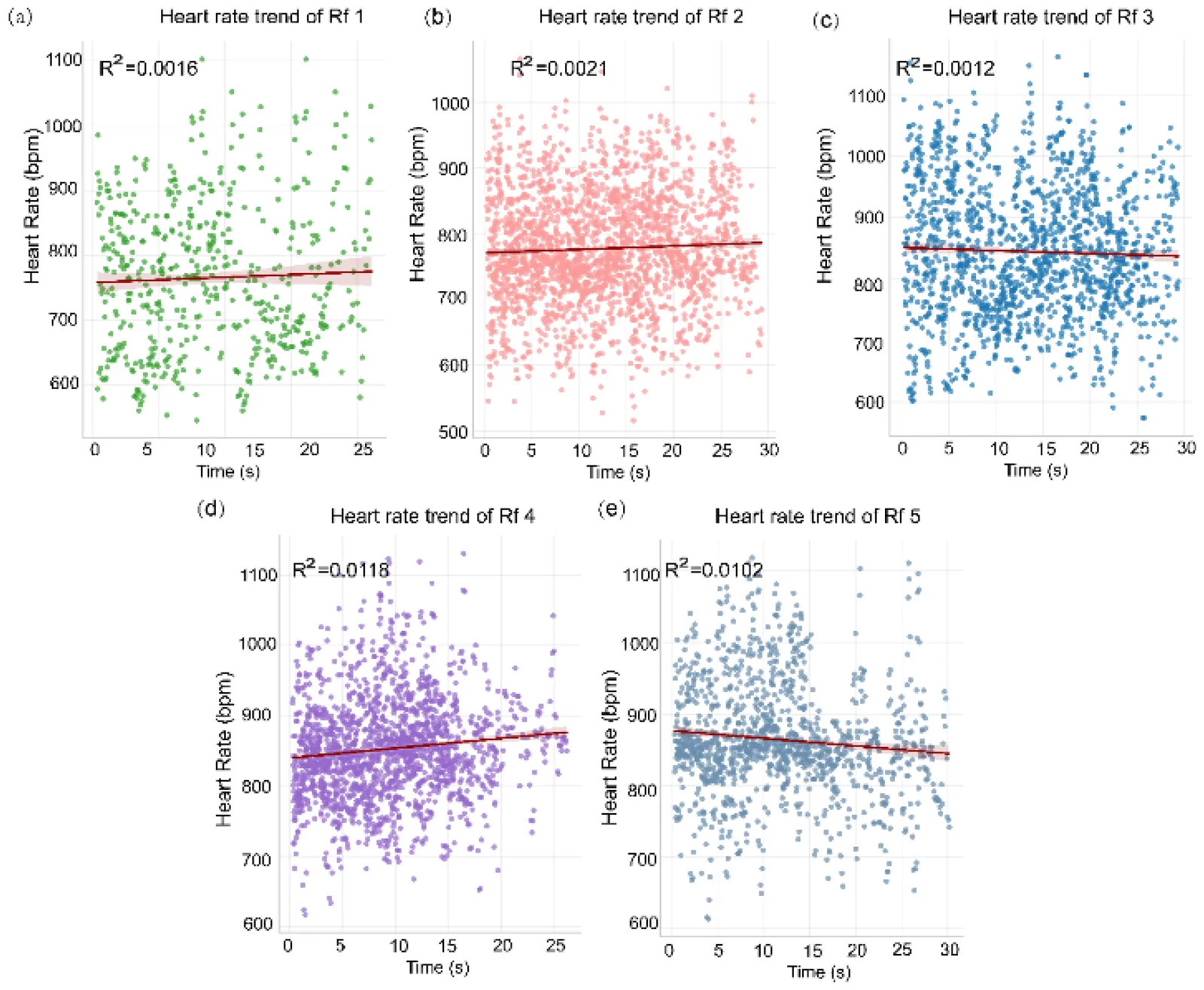
Heart rate trends over time in *R. nippon* during the experiment. Panels (**a**–**e**) correspond to individuals Rf 1–5. The red line represents the linear regression fit for heart rate over time.

During the search phase, the instantaneous heart rate of the bats fluctuated around the mean with small amplitude, and no clear time-dependent trends were observed in any individual (Figure 8). The mean heart rate during the search phase was 726.37 ± 39.52 bpm. The corresponding oxygen consumption rate, derived from heart rate, was 5.31 ± 1.29 mL/min, and the metabolic rate was 6.36 ± 1.55 kJ/h. During the approach phase, the mean heart rate of bats was 733.19 ± 34.77 bpm, with an oxygen consumption rate of 5.41 ± 1.31 mL/min and a metabolic rate of 6.49 ± 1.57 kJ/h (Table 1).

Heart rate, oxygen consumption, and metabolic rate of *R. nippon* during the approach phase increased slightly compared with the search phase, by approximately 0.94%, 1.88%, and 2.04%, respectively. Basic information on the experimental individuals, together with their heart rate, oxygen consumption, and metabolic rate, is provided in Table 1.

After controlling for individual differences using a linear mixed-effects model, the mean heart rate increased by 6.52 bpm during the approach phase compared with the search phase; however, this difference was not statistically significant (*p* = 0.12, Table 1). Heart rate remained stable throughout the experiment, exhibiting only minor fluctuations around the mean.

Linear regression analysis of instantaneous heart rate against experimental time revealed no significant correlation in any individual, with *R*^2^ values close to zero (Figure 8). Metabolic rates also did not differ significantly between the approach and search phases (*p* > 0.05, 95% CI: −2.91–24.15).

## 4. Discussion

### 4.1. Bats’ Echolocation Vocalization Regulation and Functions

This study used bat vocalization experiments to investigate the echolocation pulse spectral parameter modulation strategies of *R. nippon*. The results reveal that bats can flexibly adjust their pulse structure according to changes in prey distance. Such flexibility in echolocation substantially reduces acoustic interference, mitigates auditory masking, and facilitates the discrimination of self-emitted pulses from returning echoes [39,40].

During the search phase, bats perform broad-scale environmental scanning and initial prey detection. They emit high-energy pulses with long IPIs and extended durations, which allow coverage of greater distances. In the approach phase, bats engage in target classification and precise spatial localization. During this phase, pulse energy output is finely regulated, IPIs are sharply shortened to enhance the information update rate, and pulse duration is reduced to improve spatial resolution [41].

In the present study, as prey approached, *R. nippon* transitioned from the search phase to the approach phase. During this transition, the echolocation call rate increased significantly. Pulse duration and inter-pulse intervals were shortened, RMS amplitude and energy were markedly reduced, and peak amplitude increased significantly (Figure 4, Table S2). Notably, the call rate during the approach phase increased to 3.15 times that of the search phase (Figure 4, Table S1). This magnitude is comparable to observations in natural flight predation scenarios [26]. The result indicates that stationary bats are still able to fully express their vocal plasticity when prey approaches.

The observed trends in pulse spectral parameters of the constant-frequency echolocating *R. nippon* are consistent with previous studies on Rhinolophidae bats. They reflect a typical adaptive strategy for close-range detection. This strategy involves substantial reductions in pulse duration and inter-pulse interval, as well as significant decreases in pulse energy, peak amplitude, and RMS amplitude. During echolocation, pulse duration in *R. nippon* decreased significantly, from 38.22 ± 7.92 ms in the search phase to 24.93 ± 7.06 ms in the approach phase. Shortening pulse duration is a common adaptation that prevents overlap between emitted pulses and returning echoes. As shorter bat–prey distances, excessively long pulses could interfere with the extraction of target information [42].

IPIs decreased from 229.69 ± 142.67 ms in the search phase to 59.29 ± 36.46 ms in the approach phase. This change enhances the rate of information update per unit. It facilitates the acquisition of high-resolution target position information and meeting the requirements of close-range tracking. Reducing pulse duration and lowering echolocation pulse energy output may also reflect a long-term evolutionary strategy of bats to cope with physiological limits [22,28].

Our results on peak amplitude indicate that bats significantly increases their peak amplitude during the approach phase of echolocation, which differs from previous studies. Research on four FM bat species—*Rhinopoma microphyllum*, *Myotis myotis*, *Myotis vivesi*, and *Leptonycteris yerbabuenae*—showed that during free flight, peak frequency and call intensity are negatively correlated with IPIs. Specifically, each 1 kHz increase in peak frequency corresponds to a 7.2 ms reduction in IPI [43]. In our study, peak amplitude increased significantly from −25.38 ± 14.00 dBFS during the search phase to −22.59 ± 9.95 dBFS in the approach phase, while inter-pulse intervals decreased markedly (Figure 3).

Generally, increased peak amplitude can enhance the signal-to-noise ratio (SNR) at the onset of echoes. It also sharpens echo edges, facilitating more precise timing detection. This is advantageous for maintaining distance estimation accuracy under very short echo delay conditions. Conversely, reduced peak amplitude acts as a compensatory mechanism for shortened target distances. During the search phase, bats emit strong pulses to detect weak, distant echoes. During the approach phase under natural flight conditions, shorter distances naturally amplify returning echoes, typically resulting in a reduction in peak amplitude to prevent overly strong echoes and conserve energy. In our study, bats were stationary and could not optimize echo acquisition by adjusting flight speed or body posture. Therefore, bats may enhance the instantaneous peak of pulses to increase SNR and temporal resolution at the onset of echoes, thereby maintaining precise localization of nearby targets.

Another key finding of this study is that during the approach phase, bats primarily emit pulses as SSGs. This vocal strategy enhances information acquisition efficiency and conserves energy. During the approach phase, *R. nippon* primarily emitted doublet and triplet groups (Figure 5), with significant differences in pulse duration and RMS amplitude among pulses within each group (Figure 6 and Figure 7). These results are consistent with Stidsholt and Xia (2025), who observed in wild bats that vocalizations can be temporarily decoupled from wingbeats, and that SSGs increase the flow of sensory information during prey capture [23,44]. Rapid and stable emission of pulse SSGs allows bats to receive a series of echo snapshots from the same spatial direction. By integrating information from multiple echoes, bats can resolve the echo scene more finely. They can determine the precise position of dynamic targets, and represent the scene with higher detail [45].

Differences in pulse spectral parameters within doublet and multiplet groups also help discriminate echo sequences. This prevents confusion between pulses and echoes [46]. Within SSGs, individual pulse duration and RMS amplitude decrease as the number of pulses increases (Figure 6 and Figure 7). This pattern is consistent with Waters and Wong (2007) on the FM bat *Pipistrellus pygmaeus* [28]. In *P. pygmaeus*, reduced pulse duration and amplitude in doublet SSGs results in per-pulse energy flux density being only 40–79% of the preceding pulse. This allows the bat to gain greater benefits with only 20% additional energy expenditure. Together with previous studies, our results indicate that both CF and FM bats can flexibly modulate the energy of pulse to conserve energy. Predictable acoustic changes across foraging stages reflect a trade-off between information acquisition efficiency and behavioral investment [26].

Through dynamic modulation of echolocation pulse parameters and flexible use of SSGs, *R. nippon* achieves an optimized balance between information update rate and energy expenditure from prey search to approach. SSGs enable rapid updating of information from multiple pulse echoes. They reduce the energy expenditure of each pulse, meeting the high information update requirements of the approach phase while conserving energy. This strategy closely aligns with the detection requirements during bat foraging.

### 4.2. The Influence of Bat Echolocation Calls on Energy Metabolism and Energy-Saving Strategies

Current research on the energetic costs of echolocation has primarily focused on widely distributed FM bats. Early studies established a relationship between call rate and energy expenditure in the pipistrelle, formulated as energy expenditure (J·g^−1^·h^−1^) = 110.9 + 40.3 × pulse rate (calls/s). Based on this model, a 6 g pipistrelle bat emitting echolocation pulses at a rate of 10 call per seconds incurs a net energy cost approximately 6.79–12.2 times its basal metabolic rate [12]. In the smaller, 4 g *Thyroptera tricolor*, the energy expenditure for emitting response calls (communication calls) was found to be more than twice that of bats not producing response calls [47].

In this study, during echolocation calling, *R. nippon* exhibited an average call rate of approximately 13 calls per second in the approach phase. The maximum number of calls emitted in a single trial was 338, representing a 3.15-fold increase compared to the search phase (Figure 3, Table S1). Despite this 215.44% increase in call rate, heart rate (726.37 ± 39.52 bpm in the search phase vs. 733.19 ± 34.77 bpm in the approach phase) and metabolic rate (6.36 ± 1.55 kJ/h vs. 6.49 ± 1.57 kJ/h) did not differ significantly (Table S1). The substantial increase in call rate led to only a marginal 0.94% increase in heart rate. This stability suggests the presence of an efficient energy-saving mechanism during echolocation and implies that the vocal apparatus of *R. nippon* operates with high energetic efficiency. Waters and Wong reported that doublet emission in *Pipistrellus pygmaeus* required only an additional 20% energy to achieve greater informational gain, exemplifying a precise balance between information acquisition and energy expenditure [28]. The metabolic rate of *Tursiops truncatus* during intense vocalization can be more than 1.5 times its resting metabolic rate, while the metabolic rate during normal volume vocalization is only 1.2–1.3 times the resting metabolic rate. This demonstrates that modulations in vocal intensity and call frequency bring additional metabolic costs [14]. Based on the observed pulse parameter adjustments observed in *R. nippon* during search and approach phases, we infer that this species may flexibly regulate pulse duration, IPIs, energy, and amplitude in a stationary state. This allows them to maintain a stable heart rate and finely tune energy expenditure for sound production, thereby achieving an optimal balance between information acquisition and energy output.

Among echolocating bats, the effect of call rate on energy expenditure differs between FM and CF-FM species. In *Pipistrellus pipistrellus*, a higher stationary call rate leads to increased energy expenditure [12]. In contrast, *R. nippon* showed no significant increase in energy cost despite a substantial rise in call rate. These differences may result from body mass, and these two species belong to FM and CF-FM bats, respectively. FM bats emit broadband, short-duration pulses with multiple harmonics, suitable for foraging in open spaces, forest edges, or interior habitats. In contract, CF-FM bats produce echolocation calls with a characteristic long-duration CF component, often preceded or followed by an FM segment. These calls are primarily adapted to cluttered forest environments, and have higher fundamental frequencies than FM bats. Differences in echolocation call structure likely contribute to distinct energy expenditure patterns and suggest that CF-FM bats have evolved sophisticated acoustic energy-saving strategies.

This study provides preliminary insights into the autonomic regulation and energy-saving strategies of *R. nippon* during non-flying states echolocation, enriching our understanding of energy allocation in animal behavior. However, several questions remain to be addressed in future studies. As a CF-FM bats, *R. nippon* exhibits echolocation calls structure and vocal strategies that differ markedly from FM bats. Future studies across different echolocation types are warranted to investigate the co-evolution of call structures, ecological niche differentiation, and metabolic strategies. Furthermore, our experimental setup focused the bat’s attention directly in front of it. Future studies could increase environmental complexity by varying prey trajectories and task difficulty. They could examine how changes in prey speed or movement angle affect vocal behavior and energy expenditure, providing a more comprehensive understanding of the energetic costs and regulatory strategies of echolocation.

It should be noted that the bats’ behavioral responses to approaching prey in our experiments may differ from natural foraging conditions. In addition, the sample size of this study is relatively small (*n* = 5) for the purpose of protection of bat population, the results obtained mainly reflect the trends under the current experimental conditions. The generalization to a broader population requires careful interpretation and further confirmation through subsequent studies. Nevertheless, this study provides foundational evidence on energy expenditure associated with echolocation behavior and offers a reference framework for investigating the metabolic costs of echolocation. For future research, the three-dimensional acceleration capture of sensors could be combined to determine the energy consumption of bat echolocation vocalizations in non-flying states under field conditions. However, considering the difficulty of equipment recovery, conducting research on the energy consumption of bat echolocation behavior in a free-moving state under stationary laboratory experiments is a feasible approach.

In wildlife telemetry studies, the rule of thumb that equipment weight should not exceed 5% of the animal’s body weight is commonly adopted (Aldridge & Brigham, 1988) [48]. This rule was derived from studies on the effect of transmitter weight on flight success in insectivorous bats. In the present study, although the mass of the bat sensor reached 17.8% of body weight in individual Rf 2, and accounted for 11.8–15.5% in the remaining individuals, the bats were minimally affected by the vesper because they were fixed to avoid the high-intensity activities such as movement and flight. In addition, the threshold is often exceeded in practice, especially in the studies of small bats. Under natural conditions, in bats during night hunting, carrying young, and during pregnancy, the additional weight also exceeded 20% of their body weight [49]. During wind tunnel flight tests, bats were able to carry tags weighing 14% of their own body weight [50]. For the lightest Rf 2 individual, no abnormal states different from other individuals were recorded during the experiment, such as not emitting retrograde echolocation sound waves for detection or body struggling. There was no significant difference in heart rate of Rf 2 and Rf 3 compared to other individuals.

The acoustic pulse structure and energy allocation adjustment strategies exhibited by bats in response to energy pressure offer biological insights for achieving high information acquisition efficiency and low energy consumption in bionic sonar systems. Drawing on the auditory and echolocation mechanisms of bats, humans have made numerous attempts in the field of sonar systems. The behavioral transition of bats from the search phase to the approach phase and sensory–metabolic strategy during prey capture offers concrete design principles for low-energy, bio-inspired sonar arrays. Artificial systems could dynamically group emissions into pulse clusters and reduce per-signal amplitude upon target approach, thereby optimizing energy use without sacrificing tracking accuracy.

## 5. Conclusions

This study demonstrates that *R. nippon* balance information updating and energy expenditure through dynamic modulation of echolocation pulse parameters and flexible use of SSGs. During the search phase, bats perform broad-scale environmental sensing and initial target detection, emitting high-energy pulses with long duration and long inter-pulse intervals to cover greater distances. In the approach phase, call rate increased significantly, pulse duration and inter-pulse intervals shortened, RMS amplitude and energy of individual pulses decreased markedly, and peak amplitude increased. These changes improve target classification, spatial localization, and prey trajectory prediction. The use of SSGs in the approach phase allows for rapid information updating from multiple echo returns, while reducing the energy expenditure of per pulse.

Heart and metabolic rates remain stable even with a substantial increase in call rate, suggesting an efficient energy-saving mechanism. *R. nippon* may flexibly adjust echolocation pulse parameters to regulate echolocation energy expenditure, achieving an optimal match between information acquisition and energetic cost. This specific sensory–metabolic optimization enhances the target classification ability, spatial positioning ability, and prey trajectory prediction ability, which are the evolutionary prerequisites for bats to hunt in highly complex forest environments. Simultaneous recording and corresponding analysis of echolocation and electrocardiogram signals provides direct physiological evidence for the energetic costs of bat echolocation. It also serves as a reference for the study of the bioenergetics of animal vocal behavior and may inspire the design of low-power, bioinspired sonar systems.

## Figures and Tables

**Table 1:** Individual data of *R. nippon* and mean values of heart rate, oxygen consumption rate and metabolic during search and approach phases.

ID	Mb (g)	Gender	Category	HR (bpm)	$\dot{V}o_{2}$ (mL·min^−1^)	MR (KJ·h^−1^)
Rf 1	27.91	Female	Search	747.90 ± 13.81	6.73 ± 1.61	8.07 ± 1.93
			Approach	760.07 ± 11.62	6.98 ± 1.59	8.37 ± 1.91
Rf 2	18.93	Male	Search	711.80 ± 44.96	3.82 ± 1.17	4.58 ± 1.40
			Approach	718.48 ± 34.72	3.89 ± 1.06	4.66 ± 1.27
Rf 3	18.50	Female	Search	737.61 ± 12.97	3.99 ± 0.93	4.78 ± 1.11
			Approach	738.57 ± 10.58	3.99 ± 0.90	4.78 ± 1.08
Rf 4	27.77	Male	Search	707.66 ± 8.19	5.98 ± 1.33	7.17 ± 1.59
			Approach	711.80 ± 7.02	6.05 ± 1.32	7.25 ± 1.58
Rf 5	21.29	Male	Search	713.16 ± 4.00	4.38 ± 0.90	5.25 ± 1.08
			Approach	726.49 ± 2.68	4.55 ± 0.94	5.45 ± 1.13

Note: Search, search phase; Approach, approach phase.

## Data Availability

The data on which this study is based are available in the Supplementary Materials.

## Supplementary Materials

The following supporting information can be downloaded at https://www.mdpi.com/article/10.3390/biology15120907/s1, Table S1: The echolocation vocalization data of experimental individuals; Table S2: RMS amplitudes of doublets, triplets and multiplets in the experimental bats SSGs.

## Appendix A

**Table A1:** Results of paired sample *t*-tests on the call rates of *R. nippon* during the search and approach phases of the experiment. Asterisks indicate statistical significance.

Bat ID	N	Call Rate (Hz)		*p*-Value
		Search Phase (Mean ± SD)	Approach Phase (Mean ± SD)	
Rf 1	9	4.63 ± 2.54	11.49 ± 3.46	<0.001 ***
Rf 2	3	2.57 ± 0.71	7.73 ± 2.24	0.029 *
Rf 3	8	2.44 ± 1.36	13.15 ± 2.51	<0.001 ***
Rf 4	10	4.63 ± 1.33	14.96 ± 3.09	<0.001 ***
Rf 5	4	4.42 ± 1.15	16.25 ± 2.63	0.001 **

Note: * *p* < 0.05; ** 0.001< *p* < 0.01; *** *p* < 0.001.

**Table A2:** Comparison of echolocation acoustic spectrum parameters of *R. nippon* during the search and approach phases based on the linear mixed model.

Parameters	Estimate (*β*)	df	*t*	*p*	95% CI
Duration	−12.97	1856	−33.36	**<0.001**	−13.73~−12.21
Interval	−170.95	1859	−40.99	**<0.001**	−179.13~−162.77
RMS amplitude	−2.73	1853	−9.50	**<0.001**	−3.29~−2.17
Lg energy	−0.46	1853	−14.51	**<0.001**	−0.52~−0.40
Peak amplitude	4.20	1853	9.73	**<0.001**	3.35~5.04

Note: Bold values indicate statistically significant results when *p* < 0.001. β coefficients and confidence intervals represent the difference between the search and approach phases (Approach − Search).

**Table A3:** The random effect variance and model fitting indicators of the LMM for spectral parameters in the search and approach phases.

Response Variable	Bat ID Variance	Residual Variance	Marginal *R*^2^	Conditional *R*^2^
Duration	1.85	51.32	0.37	0.39
Interval	79.70	5914.50	0.48	0.48
RMS amplitude	98.80	28.00	0.01	0.78
Lg energy	0.99	0.34	0.03	0.75
Peak amplitude	53.20	63.10	0.03	0.47

**Table A4:** Echolocation pulses in search phase, approach phase and SSGs of *R. nippon*.

Bat ID	Trial	Search Phase	Approach Phase	Doublet	Triplet	Multiplet
Rf 1	3	155	346	105	29	11
Rf 2	3	51	244	53	26	6
Rf 3	3	75	332	22	33	9
Rf 4	3	116	337	101	31	3
Rf 5	3	59	258	65	28	4
