# Correction: Zhang et al. Energy Expenditure Optimization in the Echolocation of *Rhinolophus nippon*: Evidence from Heart Rate Stability. *Biology* 2026, *15*, 907

**DOI:** 10.3390/biology15181621

**Published:** 2026-09-15

**Authors:** Mingxin Zhang, Weihao Qi, Bo Han, Fujie Han, Hao Gu, Kangkang Zhang, Ying Liu

**Affiliations:** 1 Jilin Provincial Key Laboratory of Animal Resource Conservation and Utilization, Northeast Normal University, 2555 Jingyue Street, Changchun 130117, China; 2 Jilin Provincial International Cooperation Key Laboratory for Biological Control of Agricultural Pests, Northeast Normal University, Changchun 130117, China

## Wrong Reference

In the original publication [1], Reference 36 “Joint Committee for Guides in Metrology. Evaluation of Measurement Data: Guide to the Expression of Uncertainty in Measurement: JCGM 100:2008. Available online: https://www.bipm.org/documents/20126/2071204/JCGM_100_2008_E.pdf (accessed on 5 June 2026).” has been replaced with the following new reference: “Bishop, C.M.; Spivey, R.J. Integration of Exercise Response and Allometric Scaling in Endotherms. *J. Theor. Biol.* **2013**, *323*, 11–19.”

In the first paragraph of Section 2.5.2, following Formula (2), the reference cited as [37] in the following sentences—“where $\overset{\cdot }{V}o_{2}$ is the oxygen consumption rate (mL·min^−1^), *M*_b_ is the bat’s body mass (kg), and *f*_h_ is the heart rate (bpm). The Delta error propagation model was used to incorporate the uncertainty of the exponent into the final standard deviation calculation [37]”—has been corrected. Reference 37, previously listed as “Lighton, J. *Measuring Metabolic Rates: A Manual for Scientists*; Oxford University Press (OUP): Oxford, UK, 2008.” has been replaced with “Joint Committee for Guides in Metrology. Evaluation of Measurement Data: Guide to the Expression of Uncertainty in Measurement: JCGM 100:2008. Available online: https://www.bipm.org/documents/20126/2071204/JCGM_100_2008_E.pdf (accessed on 5 June 2026).”

In the second paragraph of Section 2.5.2, the reference cited as [37] in the following sentence: “Oxygen consumption rate $\overset{\cdot }{V}o_{2}$ was converted into energy expenditure (kJ) using the oxycalorific equivalent (kJ·L^−1^), according to the following equation from Lighton [18,37]:” has been renumbered as [38] and now refers to “Lighton, J. *Measuring Metabolic Rates: A Manual for Scientists*; Oxford University Press (OUP): Oxford, UK, 2008.”

With this correction, the order of some references has been adjusted accordingly. 

## Wrong Formula

In Section 2.5.1, Formula (1) has been corrected, as a typo, “÷” was removed due to being unnecessary.

The authors state that the scientific conclusions are unaffected. This correction was approved by the Academic Editor. The original publication has also been updated.
